# Minimally invasive video-assisted resection of a papillary fibroelastoma originating from the papillary muscle of the left ventricle: a case report

**DOI:** 10.1186/s44215-025-00216-3

**Published:** 2025-07-15

**Authors:** Hiroki Tada, Kazuma Handa, Masaro Nakae, Teruya Nakamura, Shigeru Miyagawa, Naosumi Sekiya

**Affiliations:** 1 Department of Cardiovascular Surgery, Sakurabashi Watanabe Advanced Healthcare Hospital, Osaka, Japan; 2 Department of Cardiovascular Surgery, The University of Osaka Graduate School of Medicine, Osaka, Japan

**Keywords:** Papillary fibroelastoma, Minimally invasive cardiac surgery, Papillary muscle, Video-assisted, Left ventricular tumor

## Abstract

**Background:**

Papillary fibroelastoma is a rare, benign cardiac tumor that typically originates from the cardiac valves, with papillary muscle involvement being extremely rare. However, optimal management of papillary fibroelastoma remains variable.

**Case presentation:**

A 79-year-old female with multiple comorbidities, including Parkinson’s syndrome, diabetes, and frailty, was referred to our hospital because an incidental left ventricular mass was detected during a preoperative evaluation for knee osteoarthritis. Echocardiography and computed tomography revealed a mobile, 17-mm mass in the left ventricle, possibly attached to the posterior papillary muscle. The morphological findings were suspicious for papillary fibroelastoma. The tumor was surgically resected from the papillary muscle using a three-dimensional thoracoscopy-assisted right limited-thoracotomy approach. Histopathological analysis confirmed the diagnosis of papillary fibroelastoma. Postoperatively, the patient was discharged without complications, and no recurrence was observed at the 1-year follow-up.

**Conclusion:**

This case demonstrates the feasibility and efficacy of minimally invasive video-assisted right thoracotomy for the resection of a papillary fibroelastoma originating from the papillary muscle.

## Background

Cardiac tumors are rare, with over 90% benign cases [1]. Papillary fibroelastoma (PFE) is a rare primary benign tumor that accounts for 15% of all cases in this category [1]. Most PFEs originate from the cardiac valves, although very few occur in the papillary muscles [2–4].

We report a case of PFE originating from the papillary muscle successfully resected completely using a minimally invasive right limited-thoracotomy approach.

## Case presentation

**Fig. 1:**
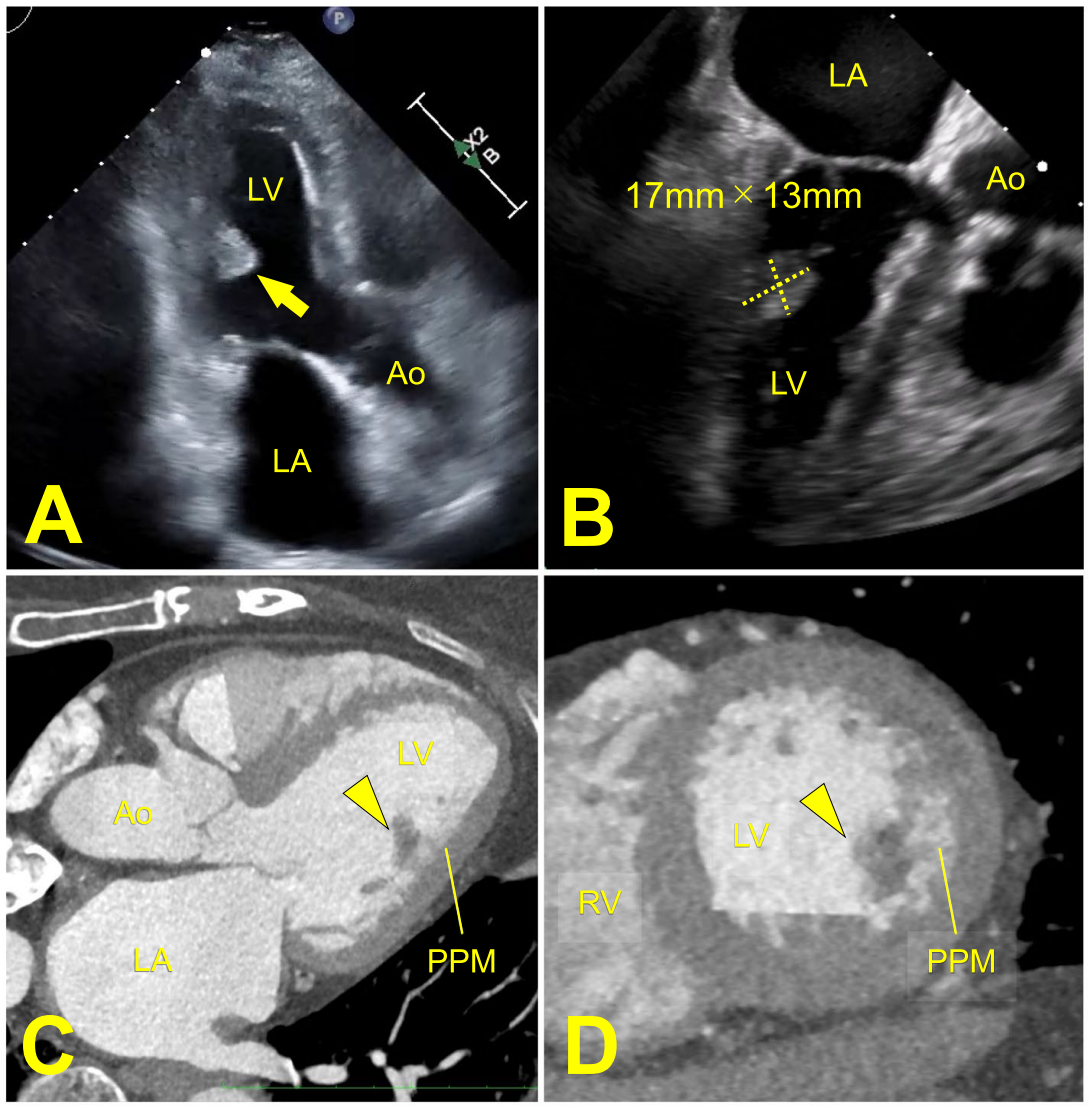
**A**, **B** Preoperative transthoracic echocardiography (**A**) and transesophageael echocardiography (**B**) reveal a highly mobile mass measuring 17 × 13 mm, attached to the posterior papillary muscle (arrow). **C**, **D** Three-lumen (**C**) and short-axis views (**D**) of cardiac computed tomography demonstrate a mass attached to the posterior papillary muscle (arrowhead). LV: left ventricle; LA: left atrium; Ao: aorta; RV: right ventricle; PPM: posterior papillary muscle

A 79-year-old woman was referred to our department after an incidental left ventricular mass was found during a preoperative examination for knee osteoarthritis. A comprehensive preoperative workup, including blood tests, chest radiography, and electrocardiogram, showed no abnormalities. Head computed tomography (CT) and magnetic resonance imaging (MRI) revealed no evidence of acute-phase cerebral infarction. Transesophageal echocardiography (TEE) revealed a mobile, 17-mm mass attached to the posterior papillary muscle of the left ventricle. There was no significant valvular insufficiency observed (Fig. 1A, B). Additionally, cardiac computed tomography revealed a mass measuring more than 10 mm attached to the posterior papillary muscle (Fig. 1C, D). Although echocardiographic findings strongly suggested papillary fibroelastoma (PFE), differential diagnoses included thrombus and other types of tumors such as myxoma. Since medical therapy was considered ineffective for preventing systemic embolization and sudden death, tumor extirpation was planned. The patient had several comorbidities, including Parkinson’s syndrome, diabetes, and severe frailty (clinical frailty score of 6). Considering the superior visualization of the operating field in the left ventricular cavity and the need to prioritize early knee arthroplasty to prevent limitations in activities of daily living (ADL), we opted for tumor resection using a minimally invasive right limited-thoracotomy.

**Fig. 2:**
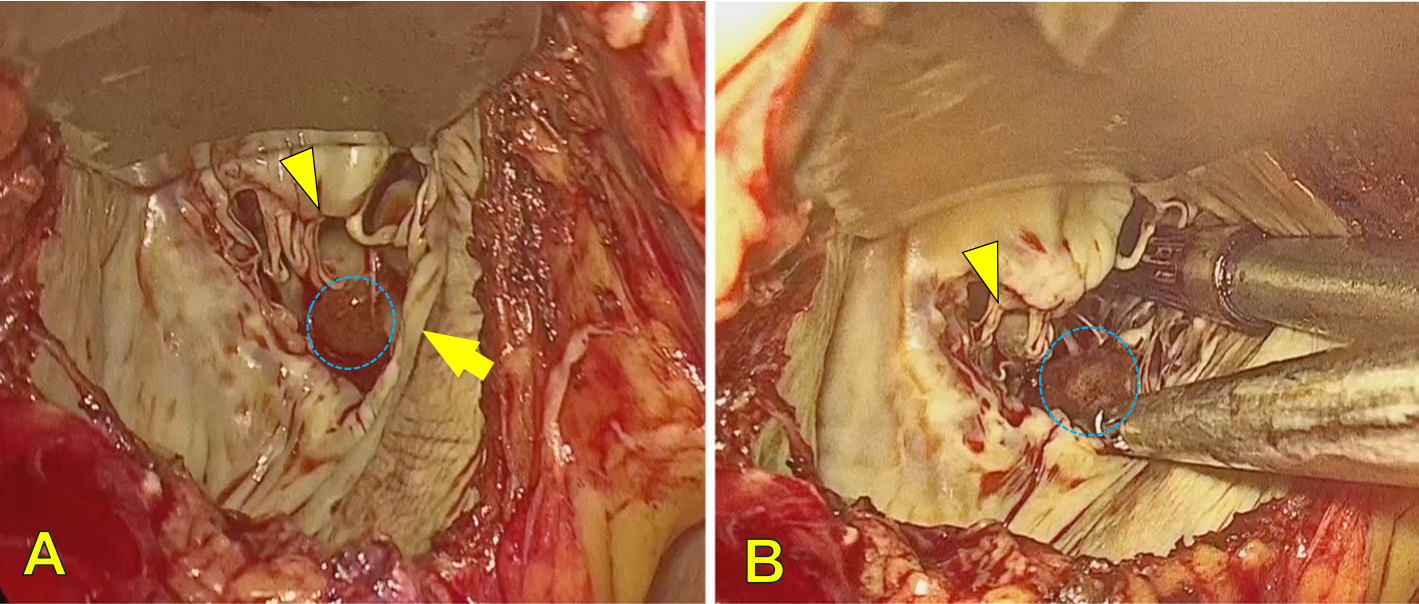
**A**, **B** Intraoperative findings: the left ventricle is visualized following a left atrial incision. The PFE is located between the anterior and posterior papillary muscles (**A**). The PFE is attached to the origin of the chordae tendineae on the medial posterior papillary muscle (**B**). (Blue circle: PFE, arrowhead: anterior papillary muscle, arrow: posterior papillary muscle) PFE: papillary fibroelastoma

**Fig. 3:**
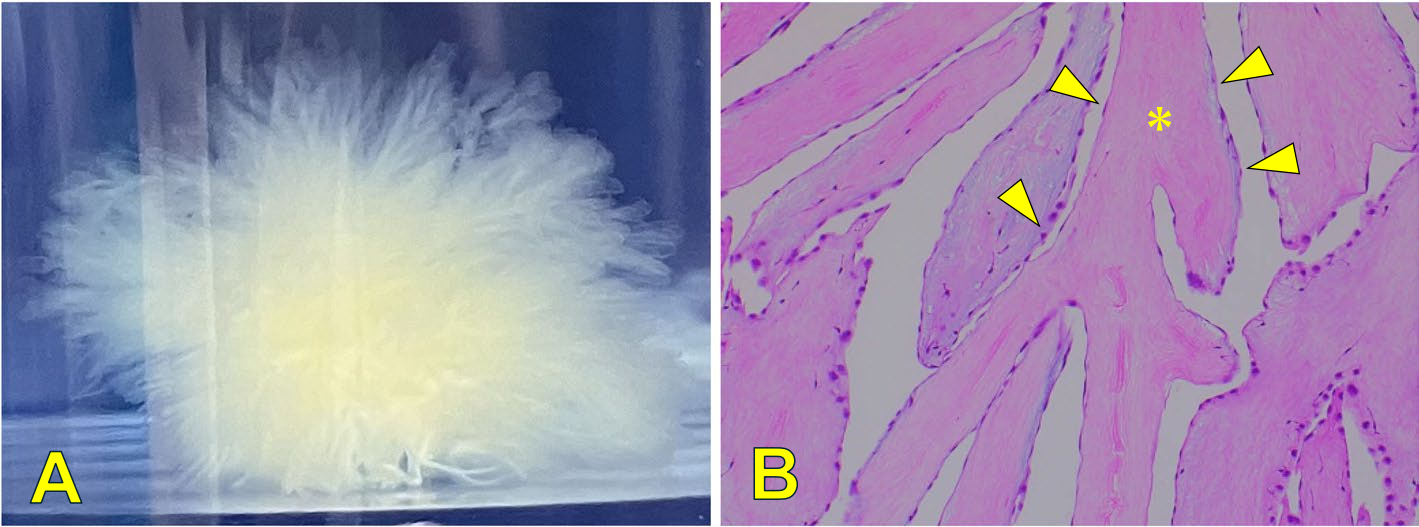
**A **Gross findings reveal a sea anemone–like structure consistent with PFE. **B** Histopathological examination shows a fronded papillary lesion, with cores of collagen (arrowhead) surrounded by elastic tissue (＊) covered with a layer of endocardial endothelium (hematoxylin and eosin staining, original magnification, × 100)

The patient was taken to the operating room, and general anesthesia was induced via endotracheal intubation. The patient was placed in the left hemi-decubitus position, and a 7-cm right anterior thoracotomy was performed in the right fifth intercostal space. Cardiopulmonary bypass was initiated by cannulating the right femoral artery and vein under mild hypothermia (32 °C). After aortic cross-clamping, antegrade cardioplegia was administered to achieve cardiac arrest. A right-sided left atrial approach provided thoracoscopic visualization of the left ventricle. A sea anemone-like mass was easily identified between the anterior and posterior papillary muscles. The tumor was attached to the lateral aspect of the posterior papillary muscle and was carefully excised along the tumor margin under thoracoscopic assistance without injury (Fig. 2A, B). Cardiopulmonary bypass was smoothly weaned off, and intraoperative TEE confirmed no residual mass and normal valve function. The cross-clamp time was 37 min, and the cardiopulmonary bypass time was 72 min. The patient was extubated 4 h postoperatively. Due to her advanced age and preexisting osteoarthritis of the knee, she underwent intensive rehabilitation to help her regain independence in normal activities. After confirming sufficient improvement in physical function, she was discharged on postoperative day 15 without complications. The resected specimen measured 15 mm and grossly appeared sea-anemone-like (Fig. 3A). Histopathological analysis revealed a central elastic core composed of collagenous tissue, consistent with PFE (Fig. 3B). The surgical margins were negative for residual tumor cells. At the 1-year follow-up, transthoracic echocardiography showed no evidence of recurrence.

## Discussions and conclusions

The majority of PFEs originate from the cardiac valves, with 44% originating from the aortic valve, 35% from the mitral valve, 15% from the tricuspid valve, and 5% from the pulmonary valve [2]. The remaining cases originate from various regions of the non-valvular endocardium, including the left ventricular septum, papillary muscle, chordae tendineae, right ventricular outflow tract, and right atrium. Among these, the occurrence of PFE originating from the papillary muscle is extremely rare [3, 4]. Differential diagnoses include vegetations, myomas, cysts, thrombi, fibromas, angiosarcomas, and primary cardiac lymphomas. While the diagnostic accuracy of PFE via TEE is high, cardiac CT and MRI can also be useful in diagnosis [5]. For malignant tumors, findings such as invasion into the pericardium on CT and the presence of systemic metastases may provide additional diagnostic clues. Furthermore, a comprehensive assessment—including clinical history, blood cultures, antiphospholipid antibodies, and lupus screening—is important for accurate preoperative evaluation [5].

Although PFEs are typically asymptomatic [6], they are clinically significant because of their potential to cause systemic embolisms, which can lead to mortality [7]. There are no established guidelines for managing PFEs; however, urgent surgical intervention is generally recommended for left-sided PFEs that are mobile or measure more than 10 mm in size [5]. While PFEs have traditionally been resected via median sternotomy, recent reports have suggested that right thoracotomy is a viable alternative for benign cardiac tumor resections, offering favorable outcomes [8–11]. Median sternotomy can limit detailed visualization inside the left ventricular cavity, and some cases require ventriculotomy depending on the tumor’s attachment site. In contrast, right thoracotomy utilized by three-dimensional thoracoscope offers a nearly panoramic view of the left ventricle and a superior surgical field. Video assistance facilitates the precise identification of residual tumor tissue within the trabeculae carneae and minimizes the risk of valvular and chordal injuries to the papillary muscle and chordae. Additionally, the right mini-limited thoracotomy approach promotes rapid postoperative recovery compared with sternotomy, facilitating a smooth transition to subsequent treatment without compromising ADL [12]. Other minimally invasive approaches, such as partial sternotomy, left mini-thoracotomy, and robotic-assisted surgery, are described below: Partial sternotomy was considered less favorable due to inferior visualization compared to video-assisted right thoracotomy, which has been shown to provide superior exposure in mitral valve surgeries [13]. Additionally, the risk of sternal osteomyelitis remains a concern with partial sternotomy [14], making right thoracotomy a safer alternative. Left mini-thoracotomy was also deemed suboptimal because a left-sided approach to the left ventricle may carry a high risk of bleeding, with limited access for hemostasis through a small incision, and would not allow for mitral valve repair if required. On the other hand, robotic-assisted resection of left ventricular tumors has been reported [15], and may represent a viable alternative in appropriate settings. In cases of complete resection of PFE, no specific postoperative treatment is generally required, as recurrence is rare [16]; however, regular follow-up with transthoracic echocardiography is recommended. In conclusion, we successfully and safely resected an extremely rare PFE originating from the posterior papillary muscle of the left ventricle using a video-assisted right mini-limited thoracotomy approach. This technique provides a clear surgical view and favorable postoperative course, even in patients with multiple comorbidities and frailty.

## Data Availability

The datasets supporting the conclusions of this article are included within the article.

## Abbreviations

CT: Computed tomography
MRI: Magnetic resonance imaging
PFE: Papillary fibroelastoma
TEE: Transesophageal echocardiography
ADL: Activities of daily living
